# Synovial Joint Fluid Metabolomic Profiles and Pathways Differentiate Osteoarthritis, Rheumatoid Arthritis, and Psoriatic Arthritis

**DOI:** 10.3390/metabo16010070

**Published:** 2026-01-12

**Authors:** Ozan Kaplan, Rositsa Karalilova, Zguro Batalov, Konstantin Batalov, Maria Kazakova, Victoria Sarafian, Emine Koç, Mustafa Çelebier, Feza Korkusuz

**Affiliations:** 1 Department of Analytical Chemistry, Faculty of Pharmacy, Hacettepe University, Ankara 06230, Türkiye; ozankaplan@hacettepe.edu.tr (O.K.); emikocc25@gmail.com (E.K.); celebier@hacettepe.edu.tr (M.Ç.); 2 Department of Propaedeutics of Internal Diseases, Clinic of Rheumatology, University Hospital Kaspela, Medical University of Plovdiv, 4002 Plovdiv, Bulgaria; rositsa.karalilova@mu-plovdiv.bg (R.K.); zguro.batalov@mu-plovdiv.bg (Z.B.); konstantin.batalov@mu-plovdiv.bg (K.B.); 3 Department of Medical Biology, Research Institute at Medical University of Plovdiv, Medical University of Plovdiv, 4002 Plovdiv, Bulgaria; mariya.kazakova@mu-plovdiv.bg (M.K.);; 4 Department of Sports Medicine, Faculty of Medicine, Hacettepe University, Ankara 06230, Türkiye

**Keywords:** synovial fluid, metabolomics, arthritis, rheumatoid, osteoarthritis, psoriatic arthritis

## Abstract

Background: Distinguishing between osteoarthritis (OA), rheumatoid arthritis (RA), and psoriatic arthritis (PsA) remains challenging despite different underlying mechanisms. Synovial fluid reflects metabolic changes within affected joints, yet comprehensive metabolomic comparisons across these conditions are limited. We aimed to identify disease-specific metabolic signatures in synovial fluid that could improve differential diagnosis and reveal therapeutic targets. Methods: We collected synovial fluid from 39 patients (20 OA, 5 RA, and 14 PsA) during routine knee arthrocentesis between January 2023 and February 2024. Following metabolite extraction, we performed untargeted metabolomic profiling using quadrupole time-of-flight liquid chromatography–mass spectrometry (Q-TOF LC/MS). Data underwent multivariate statistical analysis, including principal component analysis (PCA) and partial least squares–discriminant analysis (PLS-DA), to identify discriminatory metabolites. Results: While unsupervised analysis showed overlap between groups, supervised PLS-DA achieved clear metabolic separation. RA samples showed elevated itaconic acid, indicating inflammatory macrophage activation, and increased O-acetylserine, suggesting altered one-carbon metabolism. Hypoxanthine was decreased, which reflected severe metabolic stress. PsA exhibited the unique elevation of 4,4-dimethylcholestane and 2-oxoarginine. These metabolites have previously been unreported in this disease. OA demonstrated increased hippuric acid and indoleacetic acid, which are both gut microbiota products, supporting the gut–joint axis hypothesis. Conclusions: Each arthritis type displayed distinct metabolic fingerprints in synovial fluid. Candidate discriminatory metabolites, including gut-derived metabolites in OA and specific lipid alterations in PsA, open new diagnostic and therapeutic avenues. Given the limited RA sample size (*n* = 5), RA-related results should be viewed as exploratory and requiring validation in larger independent cohorts. These metabolites may, after rigorous validation in larger and independent cohorts, contribute to multi-metabolite biomarker panels for earlier diagnosis and to the rational design of targeted therapeutics addressing disease-specific metabolic disruptions.

## 1. Introduction

Arthritis is primarily characterized by pain, functional impairment, and reduced quality of life [1]. Osteoarthritis (OA), rheumatoid arthritis (RA), and psoriatic arthritis (PsA) are common forms of arthritis, with distinct clinical profiles and omic pathways [2]. OA is the most common form of arthritis and represents a leading cause of disability in the aging population. Approximately, one in three individuals over the age of 65 are affected, with a disproportionately higher prevalence observed among women. The global burden of OA continues to increase, driven largely by the rising prevalence of key risk factors, such as population aging and obesity [3]. RA is a chronic, systemic autoimmune inflammatory disease that primarily affects the joints and periarticular soft tissues [4]. PsA is a heterogeneous and chronic inflammatory immune-mediated disease characterized by musculoskeletal involvement, including arthritis, enthesitis, spondylitis, and dactylitis, and it typically develops in individuals with psoriasis [5]. Incidence of RA is increasing in the low- and the middle-income regions [6]. The age-standardized prevalence rate of OA increased from 6393.1 (95% uncertainty intervals of 5683.2 to 7059.5) in 1990 to 6967.3 (95% uncertainty intervals of 6180.7 to 7686.1) from 1990 to 2021, making this disease the most common form of arthritis [7]. The overall prevalence and incidence rates of PsA are 0.1–0.2% and 0.006% annually [8]. The social burden of degenerative joint diseases is increasing due to the aging of the global population and the increasing costs to health systems for their treatment. Environmental factors in combination with genetic predisposition are currently considered to be the leading factors in the pathogenesis of inflammatory joint diseases. A sedentary lifestyle with reduced physical activity is a key factor contributing to the increasing rates of metabolic syndrome and obesity. Adipose tissue plays a role as a source of pro-inflammatory adipocytokines, as well as in reducing the effectiveness of applied biological therapy. The role of the microbiome in the pathogenesis of autoimmune diseases has been proven for a long time. Synovial fluid (SF) is a critical medium for the exchange of metabolites involved in inflammatory, degenerative, and regenerative processes [9]. A comparative analysis of SF metabolomics is, therefore, essential for the early diagnosis of arthritis and may contribute to improved prognostic assessment and treatment outcomes. Recent studies [10,11,12] have focused on the metabolomic profiling of SF in different types of arthritis. Although urine and serum OA biomarkers have been identified [13,14], a specific SF biomarker and/or a panel of biomarkers for the early diagnosis of OA is currently not available [15]. The isolation of a blood sample is less invasive than SF; however, the transfer of metabolites across the synovium to the serum dilutes the potential of biomarkers [16]. SF is in direct contact with the altered or inflamed tissue, making it a promising medium for biomarker discovery in OA. A study [17] has suggested that metabolites could serve as a better tool for predicting the molecular signatures of the disease in RA patients. Another study [15] has focused on the metabolomic profiling of OA and RA patients with a small number of samples and limited patient information. This highlights the need for larger, cohort-based human OA SF studies using broad metabolomic approaches. We hypothesized that multivariate modeling of SF metabolomic profiles could reveal group-level differences among OA, RA, and PsA and help prioritize candidate discriminatory metabolite signatures that may support differential diagnosis. This study aimed to systematically profile and compare SF metabolomes from OA, RA, and PsA patients using untargeted Q-TOF LC–MS in order to identify candidate discriminatory metabolites for differential diagnosis and to explore disease-associated metabolic pathways.

## 2. Materials and Methods

### 2.1. Study Design

A cross-sectional, interdisciplinary, and multi-center clinical study was designed. The collection of patient samples was approved by the Ethics Committee at Medical University of Plovdiv (Protocol N:408.06.2022). The independent variable was disease group (OA, RA, PsA), and the dependent variables were the untargeted synovial fluid metabolic features detected by Q-TOF LC–MS.

Patients: This study included SF samples obtained from patients diagnosed with active knee OA (*n* = 20), RA (*n* = 5) and peripheral PsA (*n* = 14). Patient recruitment was performed between January 2023 and February 2024. Diagnosis and classification were made in accordance with established clinical and radiological criteria. For RA, the classification criteria of the American College of Rheumatology/European League Against Rheumatism collaborative initiative were used [18]. Additionally, the international classification criteria were used for PsA [19] and the EULAR recommendations were applied for knee OA [20]. All participants provided written informed consent, and the study protocol was approved by the relevant Ethics Committee. Patients with comorbidities known to affect systemic metabolism, such as diabetes mellitus, hyperlipidemia, renal insufficiency, and autoimmune comorbidities, were excluded. Age, sex, and body mass index (or BMI) were matched as closely as possible across study groups to minimize confounding metabolic variation.

### 2.2. Synovial Fluid Collection

Synovial fluid was collected under sterile conditions using standard arthrocentesis procedures. Briefly, knee arthrocentesis was performed with the patient in the supine position. The knee joint was slightly flexed at 15–25 degrees. The puncture was preceded by an ultrasound examination of the joint, which permitted not only the precision of the subsequent procedure, but the ultrasound-guided arthrocentesis also allowed for the aspiration of a small amount of intra-articular effusion. Knee arthrocentesis was undertaken lateral or medial to the patella, guided by ultrasonography according to the distribution of synovial fluid. The needle insertion site was marked and then the skin was disinfected. After the SF was aspirated, the needle was withdrawn. Pressure was applied for a few seconds to prevent bleeding. An expert clinician used the same arthrocentesis technique in all patents. No complications during and after the procedure was recorded. According to established EULAR recommendations, arthrocentesis was performed under ultrasound guidance for precise needle localization in the synovial fluid area. All recommendations for asepsis and antiseptics were followed, and no complications occurred after arthrocentesis. Samples were immediately placed on ice and centrifuged at 3000 rpm for 10 min at 4 °C. The cell-free supernatant was aliquoted into sterile cryotubes and stored at −80 °C until metabolomic analysis.

### 2.3. Metabolite Extraction

Samples were thawed on ice before analysis. A volume of 0.08 mL of each SF sample was transferred into a microcentrifuge tube, followed by the addition of 0.48 mL ice-cold methanol. The mixture was vortexed for 1 min and subsequently, the samples were centrifuged at 12,000 rpm for 20 min at +4 °C in a refrigerated centrifuge (Hettich Universal 320 R, Tuttlingen, Germany). After centrifugation, the supernatant was collected for metabolomic analysis. 0.2 mL aliquot of the upper methanol phase was transferred to a separate tube and evaporated to dryness using a vacuum concentrator (Labconco, CentiVap, 7310030, Kansas, KS, USA). Dried extracts were reconstituted in 0.1 mL of acetonitrile:water (1:1, *v*/*v*), and they were then vortexed and centrifuged at 12,000 rpm for 20 min at 4 °C. The final 0.08 mL supernatant was transferred into autosampler vials for Q-TOF-LC/MS analysis. Pooled quality control samples and extraction blanks were prepared for method validation and batch correction throughout the extraction process.

### 2.4. Q-TOF LC/MS Analysis

Untargeted metabolomic profiling was performed using a Q-TOF LC/MS (Agilent 6530, Santa Clara, CA, USA), which was operated in the negative electrospray ionization mode. Chromatographic separation was achieved using a reverse-phase column (2.1 × 100 mm, 2.5 µm; XBridge, Waters, Milford, MA, USA), and it was maintained at 35 °C. The autosampler temperature was set to 4 °C and the injection volume was 10 µL. The mobile phases consisted of water with 0.1% formic acid (A) and acetonitrile with 0.1% formic acid (B). The elution gradient was as follows: 0–2 min, 95% A; 2–8 min, linear gradient to 5% A; 8–10 min, held at 5% A; and 10–15 min, re-equilibration at 95% A. The flow rate was maintained at 0.4 mL/min. Mass spectrometric data were acquired over an m/z range of 75–1200.The Q-TOF mass spectrometer was operated in negative electrospray ionization (ESI) mode with the following source parameters: capillary voltage [3.5 kV], gas temperature [325 °C], drying gas flow [10 L/min], nebulizer pressure [35 psi], fragmentor voltage [175 V], and skimmer voltage [65 V]. Continuous mass axis calibration was achieved via infusion of a reference mass solution, providing lock masses at *m*/*z* [112.9856] and [1033.9881]. Data were acquired in centroid mode over an *m*/*z* range of 75–1200 with an acquisition rate of 2spectra/s. The sample injection order was randomized, and quality control samples were injected every six samples to monitor analytical stability and reproducibility. For quality assessment, pooled QC samples were injected regularly throughout the batch, and the coefficient of variation (CV%) for each feature across QC injections was calculated. Features with a QC CV% greater than 30% were excluded from multivariate analysis.

### 2.5. Data Processing and Statistics

Raw data were exported in “.mzdata” format and analyzed with MZmine 2.53. The workflow consisted of peak detection, deconvolution, isotope grouping, and alignment. Identified features were compared to extraction blanks. Metabolite identification was performed by matching accurate mass, retention time, and MS/MS fragmentation patterns against an in-house spectral library comprising 305 experimentally acquired reference spectra. This approach enables confident MSI Level 1 identification and provides higher confidence than mass-only or database-based annotations. Normalized peak intensities were determined by referencing the average total signal intensity. Peak intensities were first normalized by total ion current (TIC) (the sum of all features per sample) and then Pareto-scaled prior to multivariate analysis. Principal component analysis (PCA), partial least squares–discriminant analysis (PLS-DA), and hierarchical clustering heatmaps were extracted from normalized peak values. PLS-DA models were built using auto-scaled data and evaluated by k-fold cross-validation. Model performance was characterized by R^2^X, R^2^Y, and Q^2^ statistics. To assess whether the observed class separation exceeded random expectation and to evaluate the risk of overfitting, we performed permutation tests for each comparison (OA vs. RA, OA vs. PsA, and RA vs. PsA). The distributions of the permuted R^2^Y and Q^2^ values were compared with the original model values. Metabolite identification was performed using both an in-house spectral library and putative annotation based on accurate mass and MS/MS fragmentation. Among these, metabolites with a variable importance in projection (VIP) that scored less than 1.0 were considered statistically significant. Peak intensities were normalized by total ion current (TIC; sum of all detected features per sample) to account for global intensity differences, followed by log_10 transformation and Pareto scaling prior to multivariate analysis. PCA and PLS-DA were performed using MetaboAnalyst. Model performance was assessed by k-fold cross-validation, with R^2^X, R^2^Y, and Q^2^ statistics reported, as well as by permutation tests to evaluate whether the observed class separation exceeded random expectations. Variables with VIP scores greater than 1.0 were considered influential, in accordance with the commonly accepted threshold indicating above-average contribution to the model. For univariate analysis, one-way ANOVA was applied to log-transformed intensities to compare the OA, RA, and PsA groups, and *p*-values were adjusted for multiple testing across all detected features using the Benjamini–Hochberg false discovery rate (FDR) procedure. Unless otherwise specified, metabolites referred to as discriminatory met an FDR-adjusted significance threshold of q < 0.05. Log2 fold changes for pairwise group comparisons and the corresponding raw and FDR-adjusted *p*-values, together with VIP scores and PLS-DA loading values, are reported in the Supplementary File section. Multivariate statistical analyses were conducted using MetaboAnalyst (version 6.0).

## 3. Results

### 3.1. Demographic Data

The demographic characteristics and routine laboratory results for the study groups are presented in Table 1. Details of age, sex, and body mass index (BMI) matching are also provided.

### 3.2. Comparative Multivariate Analysis of Arthritis Metabolomes

**Figure 1:**
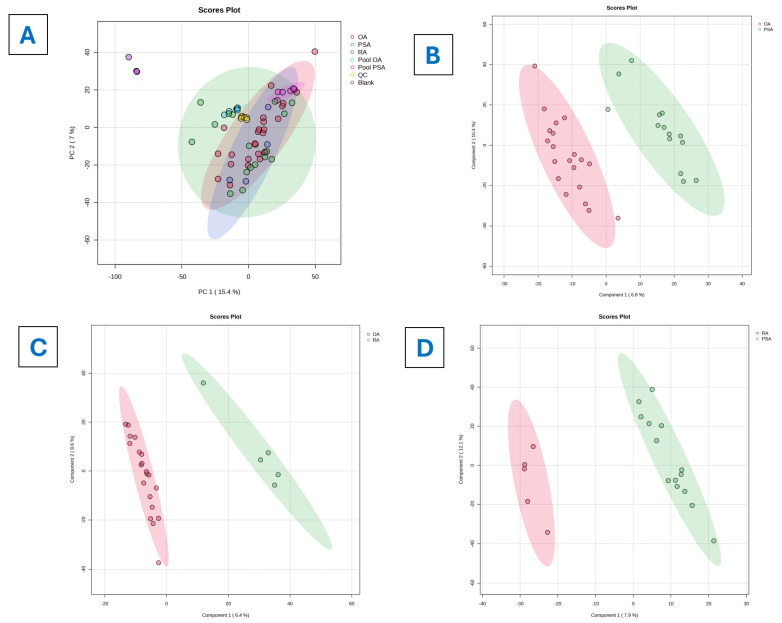
Multivariate analysis of the SF metabolomes. (**A**) Unsupervised PCA plot of all study groups. (**B**–**D**) Supervised PLS-DA plots showing clear metabolic separation between OA and PsA (**B**), OA and RA (**C**), and RA and PA (**D**).

An initial PCA (Figure 1A) revealed substantial overlap among the OA, RA, and PsA groups, with PC1 and PC2 accounting for the 22.4% of the total variance, indicating inadequate class separation by unsupervised methods. Quality control samples clustered tightly and confirmed the stability of the evaluation platform. A PLS-DA analysis subsequently demonstrated clear separation between all groups, with the PLS-DA plots showing distinct metabolic differentiation between OA and PsA (Figure 1B), OA and RA (Figure 1C), and RA and PsA (Figure 1D).

**Figure 2:**
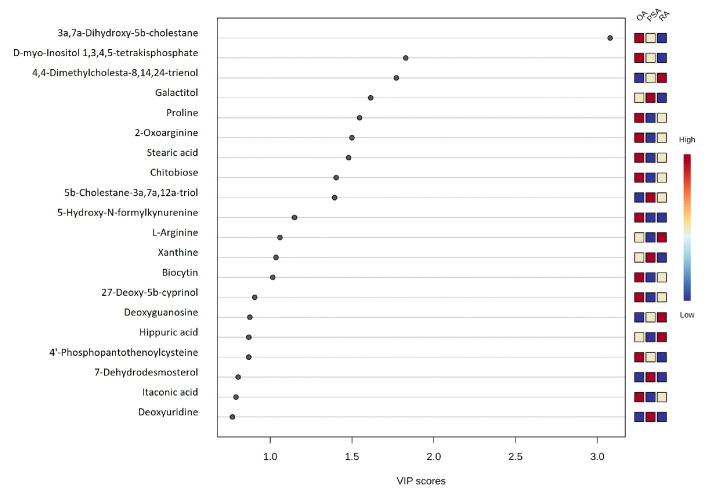
VIP score graph for the OA, RA, and PsA SF metabolomes.

A comparative analysis of SF metabolomic profiles was performed using the VIP scores among OA, RA, and PsA, revealing that metabolites such as 3a,7a-dihydroxy-5b-cholestane, D-myo-Inositol 1,3,4,5-tetrakisphosphate, 4,4-dimethylcholesta-8,14,24-trienol, galactitol, and proline exhibited the highest VIP scores, whereas hippuric acid, 4′-Phosphopantothenoylcysteine, 7-dehydrodesmosterol, itaconic acid, and deoxyuridine showed lower VIP scores (Figure 2). The values of the VIP score table are given in Supplementary Tables S1–S3.

**Figure 3:**
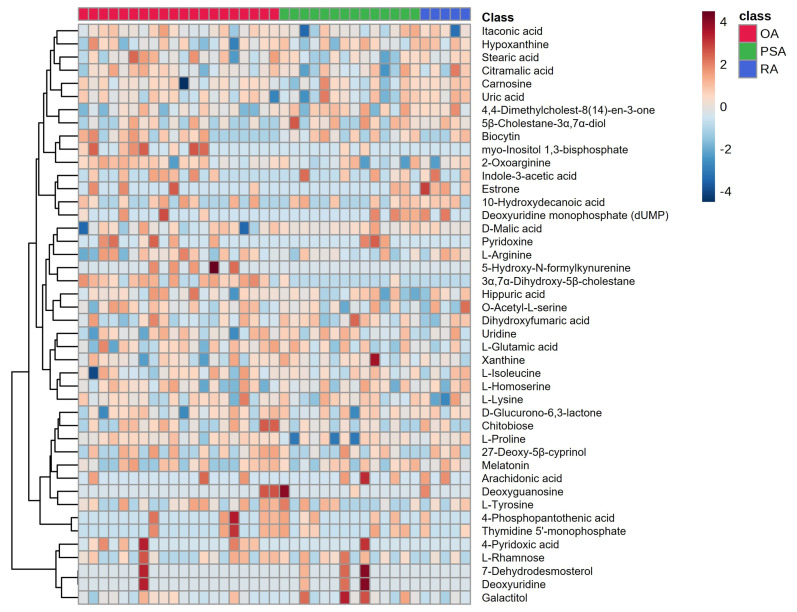
Heatmap graph for the OA, RA, and PsA SF metabolomes.

A heatmap illustrating the differential abundance of the metabolites among the OA, RA, and PsA groups is presented in Figure 3. R^2^X, R^2^Y, and Q^2^ statistics were calculated for all PLS-DA models (OA vs. RA, OA vs. PsA, and RA vs. PsA). In the cross-validated three-class model, the two- and three-component solutions yielded overall accuracies of 0.84 and 0.81, with R^2^Y values of 0.50 and 0.63 and Q^2^ values of 0.30 and 0.41, respectively. These metrics indicate a moderate model fit and modest predictive performance in the context of a small and imbalanced cohort. In contrast, the one-component model showed negligible predictive performance (Q^2^ ≈ 0.02), and adding a fourth component increased R^2^Y without improving Q^2^, suggesting the onset of overfitting. A permutation test with 1000 random permutations of the class labels confirmed that the observed class separation exceeded that expected by chance (Supplementary Figure S1); the observed PLS-DA statistic exceeded all permuted values (empirical *p* < 0.001). Taken together, these results support the use of PLS-DA as an exploratory tool for feature prioritization, while underscoring the need for cautious interpretation. These results are summarized and presented in detail in Supplementary Figure S1 and Supplementary Tables S4 and S5. To explore pathway-level alterations, pathway enrichment analysis was performed using the mummichog algorithm, which was implemented in MetaboAnalyst and based on features that were either significant after FDR correction or exhibited high VIP scores in multivariate models. Human metabolic pathway libraries (e.g., KEGG/HMDB) were used as the reference background. Enrichment *p*-values and pathway impact scores for the top pathways are reported in Supplementary Figure S2 and Supplementary Table S6. Given the small size of the RA cohort in the present study, any RA-specific metabolite signatures should be regarded as preliminary and hypothesis-generating rather than definitive disease markers. These findings require confirmation in larger, more balanced cohorts before firm conclusions can be drawn about RA-specific synovial metabolic alterations. Differential metabolites were identified between groups, and the corresponding log2 fold changes, normalized abundances, raw p-values, and FDR-adjusted q-values are provided in Supplementary Table S7.

## 4. Discussion

The multivariate analyses (Figure 1) revealed notable separation among the OA, RA, and PsA groups, suggesting genuine metabolic differences among these conditions. In the unsupervised PCA model (Figure 1A), tight QC clustering confirmed analytical reproducibility. Despite some overlap due to shared joint inflammation features, disease-specific clustering tendencies were evident. The supervised models (Figure 1B–D) provided clearer group separation. Building on this established framework, the present study aimed to extend metabolomic profiling to synovial fluid samples from OA, RA, and PsA patients. Building on existing metabolomic studies, we aimed to conduct the following: (1) explore whether previously described systemic metabolic signatures could be observed in synovial fluid, (2) investigate potential synovial fluid-specific metabolites, and (3) evaluate the capacity of metabolomic profiling to differentiate between OA, RA, and PsA within a comparative framework. The following sections present our findings in the context of the current metabolomic literature.

The metabolites with the highest VIP scores fell into several related pathways: bile-acid and cholesterol metabolism, purine and pyrimidine turnover, amino-acid modifications linked to oxidative stress, and extracellular-matrix degradation products.

Among the metabolites contributing to group separation, we observed cholestanol and cholestane derivatives, including 3α,7α-dihydroxy-5β-cholestane, 4,4-dimethylcholesta-8,14,24-trienol, and 5β-cholestane-3α,7α,12α-triol. While cholesterol-pathway metabolites have been less frequently reported in SF studies, this observation may be of interest for future investigation. These sterol intermediates belong to the same metabolic network as bile acids. Interestingly, recent serum studies by Paine et al. [21] have reported bile-acid dysregulation in PsA patients, which may suggest broader alterations in sterol metabolism, though the relationship between systemic and synovial findings remains to be established.

Purine and pyrimidine metabolites also contributed substantially to group separation. Xanthine, deoxyguanosine, and deoxyuridine all showed notable VIP scores. Kim et al. [22] previously identified hypoxanthine and xanthine as discriminators between RA and OA using gas chromatography approaches, and Anderson et al. [23] reported purine-metabolism differences in NMR-based synovial-fluid profiling. Nucleotide-pathway alterations have also been described in PsA serum samples [21]. Our data suggest that purine/pyrimidine remodeling may be informative not only for RA versus OA, but also when PsA is included in the comparison.

Several amino-acid-related metabolites showed high VIP scores in our analysis. Among these, proline demonstrated notable discriminative capacity between the OA and RA groups, though the direction and magnitude of the differences may vary across studies. Additionally, amino acid metabolism appears broadly altered in arthritis, as serum studies have consistently documented decreased branched-chain amino acids in RA patients [24]. We also detected 2-oxoarginine, an oxidative modification product of arginine, which aligns with the established evidence of mitochondrial dysfunction and oxidative stress in RA synovial tissue [25].

Itaconic acid, a TCA cycle inhibitor produced by inflammatory macrophages in model systems, may represent a metabolic mechanism relevant to RA, though direct evidence in synovial tissue macrophages is lacking [26]. Itaconic acid, detected at higher levels in RA synovial fluid, is a well-characterized product of ACOD1/IRG1-mediated macrophage activation. Although direct evidence of itaconate synthesis within human synovial macrophages remains limited, several studies have demonstrated extensive metabolic reprogramming of RA synovial macrophages, including enhanced glycolysis, altered TCA cycle flux, and activation of inflammatory metabolic nodes [26,27]. These pathways are consistent with conditions known to promote itaconate production in myeloid cells. Therefore, the elevated itaconic acid observed in RA may reflect broader macrophage-driven immunometabolic activity within the inflamed joint, although targeted validation is required to confirm its cellular origin.

We also detected hippuric acid, a gut microbiota-derived metabolite, in arthritic SF for the first time. This finding aligns with emerging gut–joint axis concepts in OA, where microbially derived metabolites, such as short-chain fatty acids, have been shown to influence disease progression through the modulation of gut barrier function and systemic inflammation [27]. Importantly, the majority of these metabolites remained significant after FDR correction, supporting the notion that the observed differences are unlikely to be solely driven by multiple testing. Metabolites not surviving FDR correction are discussed only as exploratory trends and will require confirmation in larger cohorts. Overall, the pathways highlighted in this study should be viewed as hypothesis-generating candidate pathways and not as fully validated disease mechanisms. Targeted quantitative assays, mechanistic experiments, and validation in independent cohorts will be required to confirm their involvement in OA, RA, and PsA pathophysiology.

Another important effect is the potential influence of unmeasured or uncontrolled confounders, including current medication regimens, detailed dietary habits, and the full impact of disease duration. Although major metabolic comorbidities (such as diabetes mellitus, hyperlipidemia, and renal insufficiency) were excluded and the groups were approximately matched for age, sex, and BMI, patients were receiving heterogeneous standard-of-care treatments, and synovial fluid sampling was not performed under standardized dietary conditions. These factors may have influenced both systemic and synovial metabolite levels and could contribute to residual confounding, particularly for gut-derived and inflammation-related metabolites. Depending on the osteoarthritis phenotype, patients were treated with NSAIDs; chondroprotective agents (mainly glucosamine sulfate); and, in some cases, bisphosphonates, colchicine (when crystal deposits were present), or antimalarials (in erosive OA). Patients with RA and PsA received NSAIDs; conventional disease-modifying antirheumatic drugs; and, in some cases, biologic DMARDs or targeted synthetic DMARDs. All treatments adhered to established clinical guidelines for OA, PsA, and RA. While medications and other factors are expected to influence metabolite levels, it should be noted that the patients had been on stable therapy for at least three months prior to sampling. Corticosteroids are sometimes administered after arthrocentesis; however, this does not affect metabolomic analyses, as synovial fluid collection occurs before corticosteroid administration. Future studies with medication-naïve or treatment-stratified cohorts and harmonized pre-analytical conditions (e.g., fasting status and controlled diet) will be necessary to more clearly distinguish disease-intrinsic metabolic signatures from treatment- or lifestyle-related effects.

### 4.1. Limitations

Several limitations should be acknowledged. First important limitation is the relatively small number of RA samples (*n* = 5), which reduces statistical power and may lead to underrepresentation of the metabolomic variability within the RA population. Although stringent QC procedures and cross-validation were applied to mitigate potential overfitting, the imbalance among groups warrants cautious interpretation of RA-specific findings. Recruitment of RA patients with adequate synovial effusion was limited during the study period, precluding expansion of the RA cohort. Future studies should incorporate multi-center cohorts with larger and balanced group sizes, alongside targeted validation assays, to verify the diagnostic utility of the proposed candidate discriminatory metabolites. Another important limitation is the potential influence of unmeasured or uncontrolled confounders, including current medication regimens, detailed dietary habits, and the full impact of disease duration. Although major metabolic comorbidities (such as diabetes mellitus, hyperlipidemia, and renal insufficiency) were excluded and groups were approximately matched for age, sex, and BMI, patients were under heterogeneous standard-of-care treatments and synovial fluid sampling was not performed under standardized dietary conditions. These factors may have affected both systemic and synovial metabolite levels and could contribute to residual confounding, particularly for gut-derived and inflammation-related metabolites. Future studies with medication-naïve or treatment-stratified cohorts and harmonized pre-analytical conditions (e.g., fasting status and controlled diet) will be required to more clearly distinguish disease-intrinsic metabolic signatures from treatment- or lifestyle-related effects. The untargeted metabolomics approach, while comprehensive in scope, provides semi-quantitative data and relies on putative metabolite identification based on accurate mass and MS/MS fragmentation patterns, which may lack the specificity of targeted assays. Second, the cross-sectional design precludes determining whether identified metabolites originate from systemic circulation, local synovial production, cartilage degradation, or microbial sources—longitudinal sampling or isotope tracer studies would be required to address this. Finally, these findings require validation in larger, independent, and multi-center cohorts, encompassing patients at different disease stages and treatment backgrounds before clinical translation can be considered.

Despite these limitations, this study demonstrates that metabolomics can differentiate arthritis subtypes at the molecular level, establishing a foundation for precision medicine approaches in arthritis management pending appropriate validation studies.

### 4.2. Clinical Significance

The identification of distinct metabolic signatures in synovial fluid demonstrates the potential of metabolomics to complement existing diagnostic criteria for differentiating OA, RA, and PsA at the molecular level. Notably, the discovery of candidate discriminatory metabolites, including gut-derived metabolites (hippuric acid and indoleacetic acid) in OA; itaconic acid and O-acetylserine in RA; and 4,4-dimethylcholestane and 2-oxoarginine in PsA—expands our understanding of disease-specific pathophysiology and may inform future diagnostic panels. Furthermore, the identification of metabolites linked to cholesterol metabolism, purine/pyrimidine turnover, and oxidative stress pathways suggests potential therapeutic targets for disease-specific interventions. These preliminary findings establish a foundation for precision medicine approaches in arthritis management.

## 5. Conclusions

In summary, this comprehensive comparative study indicates that OA, RA, and PsA are associated with distinct synovial fluid metabolomic profiles. These insights advance our understanding of the underlying pathophysiology of each disorder and underscore the potential for candidate discriminatory metabolites to enhance differential diagnosis, patient stratification, and future therapeutic monitoring.

## Figures and Tables

**Table 1:** Clinical and demographic characteristics of the study cohorts.

Characteristic	OA	RA	PsA
Number of patients (n)	20	5	14
Age (years), mean ± SD	61.5 ± 10.3	65.4 ± 9.5	62.8 ± 6.5
Sex (male/female)	3/17	0/5	5/9
Body Mass Index (kg/m^2^), mean ± SD	25.8 ± 2.5	23.4 ± 2.9	27.9 ± 2.9
Disease duration (years), median [IQR]	6.6 [8.5]	15 [6.0]	7.5 [5.0]
C-reactive protein (mg/L), mean ± SD	8.4 ± 10.1	12.8 ± 18.8	9.0 ± 7.7
Erythrocyte sedimentation rate (mm/h), mean ± SD	29.7 ± 14.8	28.8 ± 22.6	24.6 ± 10.3
Rheumatoid factor positive (%)	11	80	0
Anti-cyclic citrullinated peptides (positive (%)	NA	80	NA

SD: standard deviation. NA: Not available.

## Data Availability

Individual-level patient data are strictly protected and are therefore not publicly available due to legal and ethical restrictions under the Turkish Personal Data Protection Law No. 6698 (published in the Official Gazette No. 29677 on 7 April 2016), the Bulgarian Personal Data Protection Act (in force since 1 January 2002), and the EU General Data Protection Regulation (GDPR), applicable from 25 May 2018. De-identified data may be made available from the corresponding author upon reasonable request, subject to ethics approval and a data-sharing agreement.

## Supplementary Materials

The following supporting information can be downloaded at: https://www.mdpi.com/article/10.3390/metabo16010070/s1. Table S1: Component 1 value for the OA, RA, and PsA SF metabolomes; Table S2: Component 1 value for OA and PsA SF metabolomes; Table S3: Component 1 value for PSA and RA SF metabolomes; Table S4: The classification accuracy and model quality parameters (R^2^ and Q^2^) of the PLS-DA model as a function of the number of components; Table S5: The one-way ANOVA and PLS-DA results for the discriminatory metabolites among the OA, RA, and PsA groups following log transformation and FDR correction; Table S6: Pathway enrichment analysis results, including raw *p*-values and FDR-adjusted q-values; Table S7: The differential metabolites identified between groups, including fold changes, metabolite levels, and *p*-values; Figure S1: PLS-DA permutation test results; and Figure S2: Over-representation analysis (ORA) dot plot of significantly enriched metabolic pathways.

## Abbreviations

The following abbreviations are used in this manuscript:

OA	Osteoarthritis
RA	Rheumatoid arthritis
PsA	Psoriatic arthritis
Q-TOF LC/MS	Quadrupole time-of-flight liquid chromatography–mass spectrometry
PCA	Principal component analysis
PLS-DA	Partial least squares–discriminant analysis
