# Supplementary material for: Synovial Joint Fluid Metabolomic Profiles and Pathways Differentiate Osteoarthritis, Rheumatoid Arthritis, and Psoriatic Arthritis
Source: Metabolites. 2026 Jan 12;16(1):70. doi: 10.3390/metabo16010070 (PMC12844152; doi:10.3390/metabo16010070)
Supplement: Supplementary file 1 [file metabolites-16-00070-s001.zip › metabolites-3974511-supplementary.pdf]

Supplementary File 1.

Table S1. Component 1 value for OA, RA and PsA SF metabolome

**Metabolite**	**Component 1**
3a,7a-Dihydroxy-5b-cholestane	3.08
D-myo-Inositol 1,3,4,5-tetrakisphosphate	1.83
4,4-Dimethylcholesta-8,14,24-trienol	1.77
Galactitol	1.61
Proline	1.55
2-Oxoarginine	1.50
Stearic acid	1.48
Chitobiose	1.40
5b-Cholestane-3a,7a,12a-triol	1.39
5-Hydroxy-N-formylkynurenine	1.15
L-Arginine	1.06
Xanthine	1.04
Biocytin	1.02
27-Deoxy-5b-cyprinol	0.91
Deoxyguanosine	0.88
Hippuric acid	0.87
4'-Phosphopantothenoylcysteine	0.87
7-Dehydrodesmosterol	0.80
Itaconic acid	0.79
Deoxyuridine	0.77
Dihydroxyfumaric acid	0.76
D-Malic acid	0.75
Arachidonic acid	0.74
O-Acetylserine	0.73
5-Thymidylic acid	0.71
Hypoxanthine	0.67
Glutamic acid	0.66
Citramalic acid	0.58

Uridine	0.54
Indoleacetic acid	0.53
Melatonin	0.52
Rhamnose	0.32
Estrone	0.29
4-Pyridoxic acid	0.28
dUMP	0.24
L-Tyrosine	0.21
10-Hydroxydecanoic acid	0.17
Carnosine	0.09
Isoleucine	0.07
D-Glucurono-6,3-lactone	0.06
Pyridoxine	0.06
Uric acid	0.05
L-Homoserine	0.03
Lysine	0.01

Table S2. Component 1 value for OA and PsA SF metabolome

Metabolite	Component 1
4,4-Dimethylcholesta-8,14 24-trienol	2.23
3,7a-Dihydroxy-5b-cholestane	2.13
2-Oxoarginine	2.05
Stearic acid	1.54
Proline	1.53
myo-Inositol 1,3,4,5 tetrakis-P	1.48
Galactitol	1.47
L-Arginine	1.31
5b-Cholestane-3,7,12a-triol	1.28
O-Acetylserine	1.17
Dihydroxyfumaric acid	1.17
Itaconic acid	1.09
Chitobiose	1.04
5-Hydroxy-N-formylkynurenine	0.95
Uric acid	0.90
Xanthine	0.89
Isoleucine	0.82
Biocytin	0.82
Lysine	0.80
Citramalic acid	0.80
Estrone	0.76
Hippuric acid	0.75
Uridine	0.73
7-Dehydrodesmosterol	0.72

Table S3. Component 1 value for PSA and RA SF metabolome

Metabolite	Component 1
Estrone	2.72
Chitobiose	2.44
dUMP	1.77
Hypoxanthine	1.75
L-Arginine	1.68
Stearic acid	1.56
5-Thymidylic acid	1.29
Uric acid	1.26
Dihydroxyfumaric acid	1.16
Galactitol	0.98
10-Hydroxydecanoic acid	0.97
Pyridoxine	0.95
Carnosine	0.88
Rhamnose	0.86
4'-Phosphopantothenoylcysteine	0.80
Biocytin	0.77
3,7a-Dihydroxy-5b-cholestane	0.76
myo-Inositol 1,3,4,5 tetrakis-P	0.76
L-Tyrosine	0.73
Indoleacetic acid	0.72
Citramalic acid	0.71
Glutamic acid	0.69
5b-Cholestane-3,7,12a-triol	0.67
Deoxyuridine	0.61
Xanthine	0.55

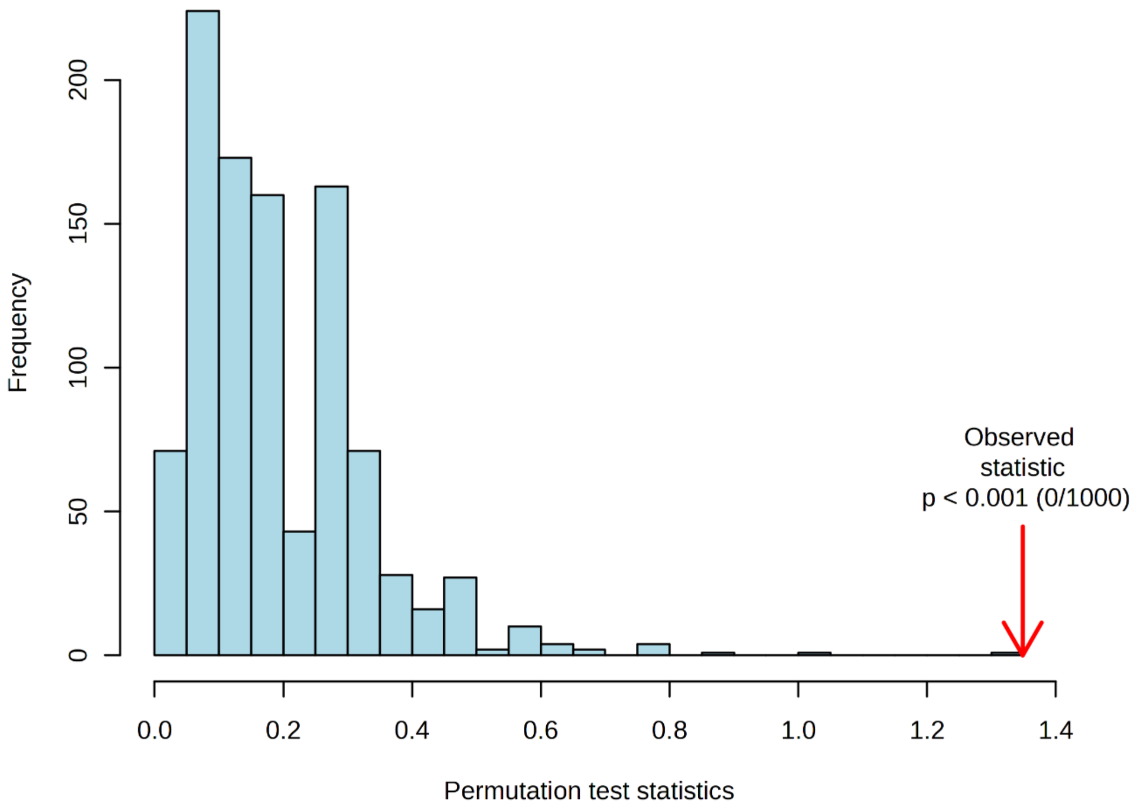

Figure S1. PLS-DA permutation test results

Table S4. Classification accuracy and model quality parameters (R^2 and Q^2) of the PLS-DA model as a function of the number of components.

Measure	1 comp	2 comps	3 comps	4 comps
Accuracy	0.69722	0.83611	0.81389	0.81389
R2	0.16339	0.50337	0.62590	0.72398
Q2	0.01668	0.30046	0.41073	0.39873

Table S5. One-way ANOVA and PLS-DA results for discriminatory metabolites among OA, RA, and PsA groups following log transformation and FDR correction.

Metabolite	False Discovery Ratio
Cortexolone	0.000
4,4-Dimethylcholesta-8,14,24-trienol	0.003
3a,7a-Dihydroxy-5b-cholestane	0.003
2-Oxoarginine	0.004
LysoSM(d18:1)	0.018
4-Bromophenol	0.020
Stearic acid	0.020
Proline	0.020
myo-Inositol 1,3,4,5-tetrakisphosphate	0.020
Galactitol	0.020
2,4,6-Tribromophenol	0.024
L-Arginine	0.027
All-trans-13,14-dihydroretinol	0.027
5beta-Cholestane-3alpha,7alpha,12alpha-triol	0.028
O-Acetylserine	0.035
Dihydroxyfumaric acid	0.035
Itaconic acid	0.040
Chitobiose	0.044
5-Hydroxy-N-formylkynurenine	0.051
9-Hydroxybenzo[a]pyrene-4,5-oxide	0.051
Uric acid	0.051
Xanthine	0.051
SN-38	0.051
Isoleucine	0.051
Biocytin	0.051
Lysine	0.051
Citramalic acid	0.051
Quinic acid	0.051
Estrone	0.051
Arachidonic acid	0.051
Hippuric acid	0.051
Deoxyguanosine	0.051
Uridine	0.051
7-Dehydrodesmosterol	0.051
Deoxyuridine	0.053
27-Deoxy-5b-cyprinol	0.053
L-Homoserine	0.055
Pyridoxine	0.066
4'-Phosphopantothenoylcysteine	0.066

D-Malic acid	0.066
Carnosine	0.066
dUMP	0.066
Benzylamine	0.066
Codeine	0.066
Glutamic acid	0.073
Indoleacetic acid	0.079

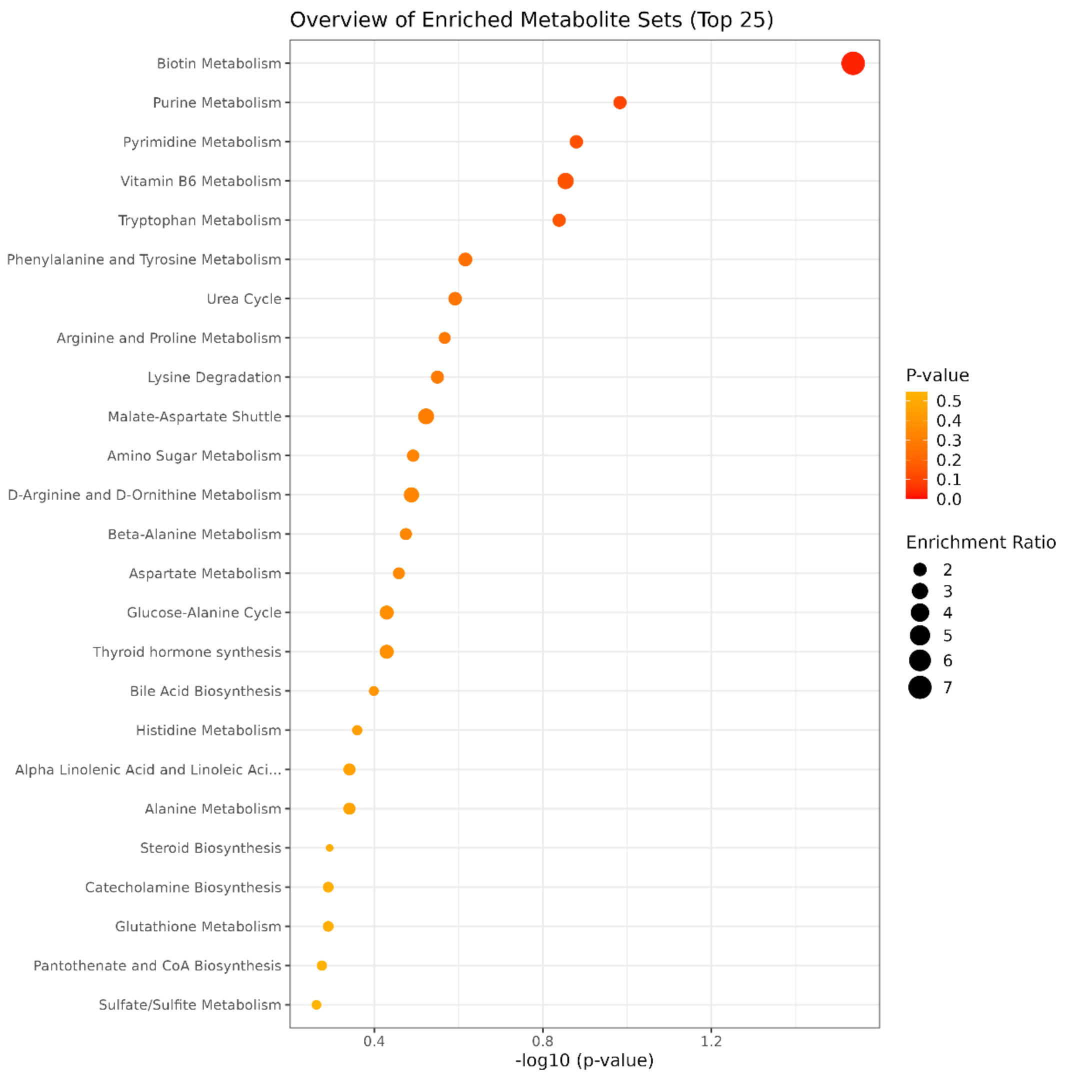

Figure S2. Over-representation analysis (ORA) dot plot of significantly enriched metabolic pathways.

Table S6. Pathway enrichment analysis results, including raw p-values and FDR-adjusted q-values.

Pathway	*p* value	False Discovery Rate
Biotin Metabolism	2.91E-02	1.00E+00
Purine Metabolism	1.04E-01	1.00E+00
Pyrimidine Metabolism	1.32E-01	1.00E+00
Vitamin B6 Metabolism	1.40E-01	1.00E+00
Tryptophan Metabolism	1.45E-01	1.00E+00
Phenylalanine and Tyrosine Metabolism	2.42E-01	1.00E+00
Urea Cycle	2.56E-01	1.00E+00
Arginine and Proline Metabolism	2.71E-01	1.00E+00
Lysine Degradation	2.82E-01	1.00E+00
Malate-Aspartate Shuttle	3.00E-01	1.00E+00
Amino Sugar Metabolism	3.22E-01	1.00E+00
D-Arginine and D-Ornithine Metabolism	3.25E-01	1.00E+00
Beta-Alanine Metabolism	3.35E-01	1.00E+00
Aspartate Metabolism	3.48E-01	1.00E+00
Glucose-Alanine Cycle	3.72E-01	1.00E+00
Thyroid hormone synthesis	3.72E-01	1.00E+00
Bile Acid Biosynthesis	3.99E-01	1.00E+00
Histidine Metabolism	4.37E-01	1.00E+00
Alpha Linolenic Acid and Linoleic Acid Metabolism	4.56E-01	1.00E+00
Alanine Metabolism	4.56E-01	1.00E+00
Steroid Biosynthesis	5.08E-01	1.00E+00
Catecholamine Biosynthesis	5.12E-01	1.00E+00
Glutathione Metabolism	5.12E-01	1.00E+00
Pantothenate and CoA Biosynthesis	5.30E-01	1.00E+00
Sulfate/Sulfite Metabolism	5.46E-01	1.00E+00
Carnitine Synthesis	5.46E-01	1.00E+00
Inositol Phosphate Metabolism	5.78E-01	1.00E+00
Androstenedione Metabolism	5.78E-01	1.00E+00
Estrone Metabolism	5.78E-01	1.00E+00
Cysteine Metabolism	6.08E-01	1.00E+00
Plasmalogen Synthesis	6.08E-01	1.00E+00
Glycine and Serine Metabolism	6.23E-01	1.00E+00
Valine, Leucine and Isoleucine Degradation	6.23E-01	1.00E+00
Mitochondrial Beta-Oxidation of Long Chain Saturated Fatty Acids	6.36E-01	1.00E+00
Folate Metabolism	6.49E-01	1.00E+00
Inositol Metabolism	6.61E-01	1.00E+00
Ammonia Recycling	6.74E-01	1.00E+00
Arachidonic Acid Metabolism	6.94E-01	1.00E+00
Androgen and Estrogen Metabolism	6.97E-01	1.00E+00
Tyrosine Metabolism	7.18E-01	1.00E+00
Nicotinate and Nicotinamide Metabolism	7.18E-01	1.00E+00
Galactose Metabolism	7.48E-01	1.00E+00

Propanoate Metabolism	7.82E-01	1.00E+00
Methionine Metabolism	7.82E-01	1.00E+00
Glutamate Metabolism	8.26E-01	1.00E+00
Warburg Effect	8.76E-01	1.00E+00

Table S7. Differential metabolites identified between groups, including fold changes, metabolite levels, and p-values.

	OA/PSA		PSA/RA		OA/RA		
**Metabolite**	**p value**	**Fold change**	**p value**	**Fold change**	**p value**	**Fold change**	**MSI Level**
2-Oxoarginine	1.80E-01	1.51	9.79E-01	-1.015	3.36E-01	1.232	Level 1
3a,7a-Dihydroxy-5b-cholestane	1.05E-01	3.22	-	-	-	-	Level 1
4,4-Dimethylcholesta-8,14, 24-trienol	1.79E-02	-2.37	7.82E-01	-1.162	1.14E-01	-1.983	Level 1
4'-Phosphopantothenoylcysteine	2.79E-01	12.07	-	1.369	-	14.023	Level 1
4-Pyridoxic acid	-	-3.58	-	-	-	-	Level 1
5b-Cholestane-3a,7a,12a-triol	2.32E-01	-3.28	3.61E-01	4.117	7.08E-01	1.307	Level 1
5-Hydroxy-N-formylkynurenine	-	-	-	-	-	-	Level 1
5-Thymidylic acid	2.06E-01	3.98	3.00E-01	1.720	4.38E-01	9.696	Level 1
7-Dehydrodesmosterol	-	1.15	-	-	-	-	Level 1
Arachidonic acid	6.04E-01	-1.53	5.68E-01	1.971	5.50E-01	42.177	Level 1
Biocytin	1.13E-01	3.44	7.70E-01	-1.349	5.51E-01	1.052	Level 1
Carnosine	6.92E-01	1.06	3.37E-01	-1.271	7.50E-02	1.448	Level 1
Chitobiose	1.60E-01	9.44	5.10E-02	-2.910	5.08E-01	3.290	Level 1
Citramalic acid	3.98E-01	1.06	3.70E-01	-1.125	5.52E-01	1.158	Level 1
Deoxyguanosine	5.15E-01	-4.53	-	15.565	-	2.878	Level 1
Deoxyuridine	-	1.21	-	-	-	-	Level 1
D-Glucurono-6,3-lactone	8.17E-01	1.05	9.35E-01	1.023	8.03E-01	2.641	Level 1
Dihydroxyfumaric acid	3.65E-01	-1.31	4.69E-01	1.429	8.39E-01	-1.444	Level 1
D-Malic acid	7.21E-01	-1.07	5.69E-01	1.128	8.38E-01	1.792	Level 1

D-myo-Inositol 1,3,4,5 tetrakisphosphate	1.88E-01	10.83	-	-	-	-	Level 1
dUMP	5.35E-01	2.20	2.16E-01	-1.724	8.70E-01	-1.378	Level 1
Estrone	3.20E-01	2.63	5.27E-01	-2.925	8.91E-01	2.479	Level 1
Galactitol	5.97E-02	-5.41	3.10E-01	9.239	2.73E-01	1.410	Level 1
Glutamic acid	5.52E-01	1.17	4.04E-01	1.388	2.81E-01	15.023	Level 1
Hippuric acid	3.51E-01	1.73	6.02E-01	-1.294	7.34E-01	-1.296	Level 1
Hypoxanthine	5.65E-01	-1.08	2.56E-01	-1.210	1.41E-01	-1.664	Level 1
Indoleacetic acid	5.74E-01	-1.49	9.41E-01	-1.079	4.76E-01	3.581	Level 1
Isoleucine	9.16E-01	1.01	7.73E-01	-1.041	8.84E-01	-1.396	Level 1
Itaconic acid	3.76E-01	1.12	9.63E-01	-1.011	5.59E-01	1.034	Level 1
L-Arginine	1.94E-01	1.24	1.65E-01	-1.299	8.31E-01	-1.277	Level 1
L-Homoserine	9.97E-01	-1.00	8.61E-01	-1.041	8.67E-01	-1.228	Level 1
L-Tyrosine	2.63E-01	-1.84	3.51E-01	4.277	2.94E-01	8.614	Level 1
Lysine	9.63E-01	-1.01	7.28E-01	1.095	7.42E-01	-1.316	Level 1
Melatonin	1.84E-01	1.54	8.61E-01	1.087	3.02E-01	1.409	Level 1
O-Acetylserine	2.06E-01	1.26	2.45E-01	-1.540	5.13E-01	-1.102	Level 1
Proline	5.66E-01	1.08	9.85E-01	-1.004	6.90E-01	1.952	Level 1
Pyridoxine	5.66E-01	-1.46	-	-	-	-	Level 1
Rhamnose	7.62E-01	1.32	4.70E-01	4.698	5.86E-01	5.128	Level 1
Stearic acid	1.03E-01	1.58	1.76E-01	-1.340	6.75E-01	116.553	Level 1
Uric acid	8.92E-01	1.02	2.55E-01	-1.245	8.49E-02	-2.429	Level 1
Uridine	5.18E-01	-1.09	6.84E-01	1.083	9.76E-01	-1.173	Level 1
Xanthine	2.74E-01	-5.69	5.40E-01	9.666	3.55E-01	1.697	Level 1
